# Identification of 1,6-hexadecanediol and its wax diesters in chloroplasts of *Nicotiana benthamiana*

**DOI:** 10.1007/s00425-025-04833-8

**Published:** 2025-10-09

**Authors:** Regina Wehler, Nina Hoppe, Katharina Gutbrod, Viktoria V. Zeisler-Diehl, Helga Peisker, Nicolas Gisch, Per Hofvander, Ida Lager, Lukas Schreiber, Peter Dörmann

**Affiliations:** 1 Institute of Molecular Physiology and Biotechnology of Plants (IMBIO), University of Bonn, Karlrobert-Kreiten-Straße 13, 53115 Bonn, Germany; 2 Division of Bioanalytical Chemistry, Research Center Borstel, Leibniz Lung Center, Parkallee 4a, 23845 Borstel, Germany; 3 Department of Plant Breeding, Swedish University of Agricultural Science, Växtskyddsvägen 1, 23456 Alnarp, Sweden; 4 Institute for Cellular and Molecular Botany (IZMB), University of Bonn, Kirschallee 1, 53115 Bonn, Germany

**Keywords:** Wax Diesters, 1,6-Hexadecanediol, *Nicotiana benthamiana*, Chloroplast, Esterase/Lipase/Thioesterase, Phytyl Ester Synthase

## Abstract

**Main conclusion:**

Expression of the *Arabidopsis* phytyl ester synthase PES2 in *Nicotiana benthamiana* chloroplasts resulted in the accumulation of fatty acid phytyl esters and wax diesters containing the novel alkanediol 1,6-hexadecanediol.

**Abstract:**

Dihydric long-chain alcohols carrying two hydroxyl groups are low abundant in plants and are mostly found in the cutin layer of leaves or the suberin of roots. Transient expression of the phytyl ester synthase PES2 from *Arabidopsis thaliana* in *Nicotiana benthamiana* resulted in the accumulation of fatty acid phytyl esters (FAPEs) and of a new lipid class that was identified as wax diesters of 1,6-hexadecanediol, carrying mostly lauric acid (12:0) and myristic acid (14:0) residues. The synthesis of FAPE and wax diesters was only observed when PES2 was targeted to the chloroplasts, in agreement with the finding that both FAPE and wax diesters are chloroplast-localized. The accumulation of wax diesters following PES2 expression demonstrates that the dihydric long-chain alcohol, 1,6-hexadecanediol, is an authentic compound produced in *N. benthamiana* chloroplasts. 1,6-Hexadecanediol in *N. benthamiana* is likely synthesized by a chloroplast-localized fatty acid reductase (FAR) in combination with a P450 monooxygenase. PES2-mediated acylation might result in chloroplast-trapping of 1,6-hexadecanediol which is possibly an intermediate in the biosynthesis of functional compounds in leaves or other plant organs.

**Supplementary Information:**

The online version contains supplementary material available at 10.1007/s00425-025-04833-8.

## Introduction

Plants produce a variety of wax esters of long-chain alcohols and fatty acids. Such wax esters are commonly found in the wax layer of the cuticle on the surface of the aerial parts of the plant (Li-Beisson et al. 2013). Cuticular wax esters typically contain long-chain alcohols and very long-chain fatty acids. The synthesis of cuticular waxes starts at the endoplasmic reticulum (ER) of epidermal cells where plastid-derived fatty acids are elongated and converted into alkanes, aldehydes, and primary and secondary alcohols (Li-Beisson et al. 2013; Yeats and Rose 2013). In *Arabidopsis*, the esterification of primary alcohols to very long-chain fatty acids is predominantly catalyzed by the acyltransferase WSD1 (Li et al. 2008). Wax esters are subsequently exported to the cuticle on the plant surface. The load and composition of wax esters varies among species and between different plant organs (Li-Beisson et al. 2013). In *Arabidopsis*, for example, the amount of wax esters on the leaves is extremely low, whereas on stems, it is considerably higher. *Arabidopsis* stem wax esters consist of total chain lengths ranging from 38 to 52 carbon atoms (Lai et al. 2007). Wax esters are also found in the seed oil of some plants, e.g., jojoba (*Simmondsia chinensis*) where they are produced by the jojoba-type wax synthase (Greene and Foster 1933; Lardizabal et al. 2000).

Furthermore, wax diesters that are composed of an alkanediol and two esterified fatty acids are found in the cuticular waxes of some plant species. For example, wax diesters of 1,ω-alkanediols are present in wheat, oat, and rye (Tulloch 1971; Tulloch and Hoffman 1974). Non-esterified alkanediols have been found in cuticular waxes of conifer needles, leaves of dicotyledonous plants, and fern fronds (Franich et al. 1979; Jetter et al. 1996; Jetter and Riederer 1999; Wen et al. 2006). In *Arabidopsis*, free alkanediols have been found in stem waxes, and esterified 1,ω-alkanediols are components of the seed suberin polyester (Molina et al. 2006; Wen and Jetter 2009). The genes involved in wax diester formation in plants remain enigmatic.

Fatty acid esters with the isoprenoid alcohol phytol, which is derived from chlorophyll breakdown, are produced in the chloroplasts (Grob and Csupor 1967; Gellerman et al. 1975; Cranwell et al. 1985; Buchanan et al. 1996; Rontani et al. 1999). Fatty acid phytyl esters (FAPEs) accumulate in *Arabidopsis* chloroplasts of leaf mesophyll cells during senescence and nitrogen deprivation, and the major molecular species are 16:3-phytol, 10:0-phytol, 12:0-phytol, and 14:0-phytol (Ischebeck et al. 2006; Gaude et al. 2007). Two *Arabidopsis* genes with sequence similarity to the esterase/lipase/thioesterase family of acyltransferases (ELT), Phytyl Ester Synthase 1 (PES1) and 2 (PES2), are involved in FAPE synthesis (Lippold et al. 2012). The two PES proteins were localized to the plastoglobules of chloroplasts (Vidi et al. 2006; Ytterberg et al. 2006; Lippold et al. 2012). The hydroxyl groups of xanthophylls in the chloroplasts can also be esterified with fatty acids. Fatty acid xanthophyll esters accumulate in fruits of *Physalis,* apples, pepper, maize, and wheat grains (Kuhn and Wiegand 1929; Knee 1988; Janick-Buckner 1999; Hornero-Méndez and Mínguez-Mosquera 2000; Paznocht et al. 2018). Among the esterified xanthophylls from apple, monoesters of neoxanthin and violaxanthin and diesters of lutein, violaxanthin, and neoxanthin were identified, mainly containing 16:1 and 18:1 and to a lower extent 12:0, 14:0, and 18:0 (Knee 1988; Bunea et al. 2014). The green microalga *Haematococcus pluvialis* accumulates astaxanthin only in its esterified form (Schoefs et al. 2001). The petals of plants like marigold (*Tagetes*) and tomato also contain high amounts of fatty acid xanthophyll esters (Gau et al. 1983; Ariizumi et al. 2014). The flowers of the tomato mutant *pale yellow petal 1* (*pyp1*) have whitish petals which lack fatty acid xanthophyll esters. The underlying protein, PYP1, shares 58% amino acid identity with PES1 (Ariizumi et al. 2014). The expression of *Arabidopsis* PES1 or PES2 in the tomato *pyp1* mutant partially restored the petal discoloration and xanthophyll ester content (Fu et al. 2025). Transgenic *Arabidopsis* plants overexpressing the β-carotene oxygenase from *H. pluvialis* accumulate free and esterified forms of keto-carotenoids (Stålberg et al. 2003) indicating that *Arabidopsis* contains the enzymes capable of xanthophyll esterification. Xanthophyll esters have not been detected in wild-type *Arabidopsis* leaves, but in tissue-cultured etiolated callus (Fu et al. 2025). While callus of wild-type *Arabidopsis* contains xanthophyll mono-, di-, and triesters, the callus of *pes1 pes2* double mutant plants is devoid of xanthophyll esters (Fu et al. 2025). Therefore, acyltransferases of the ELT family, including PES1, PES2, and PYP1, can accept phytol and xanthophylls as substrates.

Overexpression of *Arabidopsis* PES2 together with fatty acyl-CoA reductases (FAR) in a biotechnological approach resulted in the accumulation of wax esters in transiently transformed leaves of *Nicotiana benthamiana* (Aslan et al. 2014). The accumulation of wax esters was dependent on the co-expression with a FAR enzyme, indicating that PES2 can synthesize wax esters with different alcohol groups. Here, we present the analysis of the additional products of PES2 expression in *N. benthamiana*. Surprisingly, PES2-expressing leaves accumulated not only esters with monohydric alcohols like phytol but also diesters with 1,6-hexadecanediol, a dihydric alcohol not previously detected in *N. benthamiana*.

## Materials and methods

### Chemicals and reagents

1,2-Hexadecanediol and 1,16-hexadecanediol were purchased from TCI Chemicals (Eschborn, Germany). 1,6-Hexadecanediol was synthesized by Larodan (Solna, Sweden). Heptadecanoic acid (17:0), octadecane-1-ol (18:0ol), and sterol esters (16:0-cholesterol, 16:1-cholesterol, 18:0-cholesterol, and 18:1-cholesterol) were purchased from Merck/Sigma-Aldrich (Taufkirchen, Germany). FAPEs (17:0-phytol), wax esters (17:0–18:0ol), and 1,16–16:0diol diesters have been chemically synthesized via the formation of acyl chlorides (Gellerman et al. 1975). 1,2–16:0diol diesters have been synthesized via the formation of anhydrides (Kanda and Wells 1981). After the reactions were completed, the fatty acid phytyl esters, wax esters, and diesters were extracted with hexane and purified by thin-layer chromatography (TLC).

### Transient transformation of *N. benthamiana* leaves

*N. benthamiana* plants were grown on soil containing 30% (v/v) vermiculite under long-day conditions (16 h light, 8 h dark) at 25 °C, with 55% humidity and a light intensity of 250 µmol m^−2^ s^−1^. The binary construct harboring the full-length PES2 sequence has been described before (Aslan et al. 2014). The N-terminally truncated sequences of m1PES (excluding amino acids 1–64) and m2PES (excluding 1–93) lack putative targeting sequences according to different prediction programs (https://suba.live/; https://aramemnon.botanik.uni-koeln.de/) (Lippold et al. 2012). The α-fold software (www.uniprot.org/) predicts a non-structured domain for the N-terminal amino acids 1–69 of PES2, typical for transit peptides, while the C-terminal region carries a conserved α-helix. Furthermore, PES2 was localized to the plastoglobules in chloroplasts by proteomics (Ytterberg et al. 2006; Lundquist et al. 2012). The proteomics experiments with isolated chloroplasts or plastoglobules identified peptides covering the sequence of amino acids 68–701 (C-terminus), indicating that the N-terminal part of PES2 was processed prior to chloroplast uptake (www.uniprot.org/; https://peptideatlas.org/). We have shown experimentally that PES2 is taken up by isolated pea chloroplasts and is processed by removal of a ~ 10 kDa sequence (Lippold et al. 2012). While the exact length of the transit peptide remains unclear, these results indicate that PES2 carries an N-terminal chloroplast targeting sequence and that removal of this sequence presumably mis-targets the protein to the cytosol, where it might associate with membranes such as the ER due to the presence of a predicted transmembrane domain (https://aramemnon.botanik.uni-koeln.de/).

The truncated sequences m1PES2 and m2PES2 were amplified by PCR from PES2 cDNA using the primers mPES2attB1 (GGG GAC AAG TTT GTA CAA AAA AGC AGG CTT CAA CAA TGG CGA AGG TGG TGG AGA ATC) and mPES2attB2 (GGG GAC CAC TTT GTA CAA GAA AGC TGG GTC TTA GAG ATC AAA CGT TGG AAT TTC AG) for m1PES2, and the primers mPES2.2attB1 (GGG GAC AAG TTT GTA CAA AAA AGC AGG CTT CAA CAA TGA GAG AGT TCG TCG GAG ATG GAG) and mPES2attB2 (see above) for m2PES2 with Phusion polymerase. The fragments were recombined into pDONR221 using BP clonase to generate pENTRY-m1PES2 and pENTRY-m2PES2. The fragments were then recombined into pXZP393 using LR clonase to generate p35S-m1PES2 and p35S-m2PES2 which were cloned into *E. coli* TOP10 (Thermo Fisher). Finally, the constructs were transferred into *Agrobacterium tumefaciens* GV3101. Fresh leaves of four-week old *N. benthamiana* plants were infiltrated using a syringe with *A. tumefaciens* cells (GV3101-pMP90) harboring the respective constructs, cells containing the viral suppressor P19 construct, and cells containing a green fluorescent protein construct (GFP) (Wood et al. 2009). After 4–7 days, gene expression was detected by observing the GFP signal with a fluorescent lamp. Leaf areas showing expression were excised and used for lipid isolation.

### Chloroplast isolation

Chloroplasts were isolated from leaves as described (Hilt-brunner et al. 2001; Vidi et al. 2006). Briefly, infected leaves of *N. benthamiana* plants were kept in darkness for 18 h to avoid starch accumulation prior to chloroplast isolation. The GFP-expressing areas of at least 24 infiltrated leaves were collected into ice cold water. Leaves were homogenized in 50 ml HB buffer (450 mM sorbitol, 20 mM Tricine-KOH, pH 8.4, 10 mM EDTA, 10 mM NaHCO_3_, 1 mM MnCl_2_) using a tissue blender with rotating knives at 15,000 rpm for 10 s. The mixture was filtered through Miracloth and centrifuged for 3 min at 2,500 g at 4 °C. The pellet was suspended in RB buffer (300 mM sorbitol, 20 mM Tricine-KOH, pH 7.6, 2.5 mM EDTA, 5 mM MgCl_2_), loaded onto a Percoll step gradient (40% and 85% in RB buffer) and centrifuged for 30 min at 2,500 g at 4 °C. Chloroplasts were harvested from the interphase and washed with RB buffer.

### Lipid extraction and purification

For the extraction of FAPE, sterol esters, wax esters, and wax diesters, leaf material was frozen in liquid nitrogen and homogenized for 30 s at 6,000 g in the Precellys homogenizer (Bertin Instruments). Internal standards were added (1 nmol each; 17:0-phytol for FAPEs and wax diesters; 17:0–18:0ol for wax esters; 16:0-cholesterol, 16:1-cholesterol, 18:0-cholesterol and 18:1-cholesterol for sterol esters). Lipids were extracted with 500 μl diethyl ether and 250 μl 1 M KCl/0.2 M H_3_PO_4_. After vortexing, the samples were centrifuged for 3 min at 5,000 g. The extraction with diethyl ether was repeated twice and the lipid extracts were combined.

FAPE, sterol esters, wax esters, and wax diesters were purified by solid-phase extraction (Tulloch and Hoffman 1974; vom Dorp et al. 2015). The solvent of the lipid extract was evaporated, the lipids dissolved in hexane, and loaded onto the silica column (Chromabond silica, Macherey & Nagel, equilibrated in hexane). FAPE, sterol esters, and wax esters were eluted with 3 ml hexane/diethyl ether (99:1, v/v). Wax diesters were eluted with 3 ml hexane/diethyl ether (98:2, v/v) (Tulloch and Hoffman 1974). Lipid extracts containing plant-derived or synthetic wax diesters were separated by TLC on Silica 60 Durasil-25 plates (Macherey & Nagel) with hexane/diethyl ether/acetic acid (90:10:1, v/v/v). Lipid bands on TLC plates were stained with primuline and lipids were visualized under UV light.

### Alkanediol measurements by gas chromatography-mass spectrometry (GC–MS)

Purified wax diesters were cleaved in acidic (1 N methanolic HCl, 30 min, 80 °C) or alkaline (0.6 M methanolic KOH, 4 h, 40 °C) conditions. The free hydroxyl groups of the diols were silylated with *N*,*O*-bis(trimethylsilyl)trifluoroacetamide (BSTFA) at room temperature overnight. BSTFA was evaporated and the lipids were dissolved in hexane. Lipids were analyzed on an Agilent 5975C inert GC–MS instrument using an Agilent HP-5MS capillary column (30 m, 0.32 mm diameter, 0.1 µm film thickness) with the following temperature program: hold initial 70 °C for 5 min, increase 5 °C min^−1^ to 310 °C, hold for 1 min; equilibrate at 70 °C.

### Lipid analysis by quadrupole time-of-flight mass spectrometry

Lipids were quantified by direct infusion quadrupole time-of-flight mass spectrometry (Agilent 6530 Q-TOF MS/MS) with internal standards. Lipids were dissolved in chloroform/methanol/300 mM ammonium acetate (300:665:35, v/v/v) and directly infused by nanospray using an Agilent HPLC-Chip Cube MS interface at a flow rate of 1 μl min^−1^. The Q-TOF MS/MS parameters were previously described (Gasulla et al. 2013). The collision energy for fragmentation was optimized for each lipid class. Lipid species were quantified by neutral loss or by product ion scanning (for FAPE, wax esters, and wax diesters, see Tables S1–S3; for phospholipids, galactolipids, and sterol esters (Gasulla et al. 2013; Wewer and Dörmann 2014)). Raw data were corrected for isotopic overlap, and absolute amounts were calculated relative to the internal standards.

The hexadecanediols released after hydrolysis of wax diesters were dissolved in acetonitrile and separated by liquid chromatography-mass spectrometry LC–MS (Agilent 6530 Q-TOF) on a reversed-phase column (Eurospher II C8, Knauer, Berlin) using a gradient of solvent A (H_2_O/acetonitrile/formic acid, 63:37:0.02, v/v/v) and solvent B (2-propanol/acetonitrile, 50:50, v/v) by electrospray ionization in the positive mode (Kortz et al. 2013).

### Lipid X analysis by nuclear magnetic resonance (NMR) spectroscopy

NMR spectroscopic measurements of lipid X were performed in CDCl_3_ at 300 K on a Bruker Avance^III^ 700 MHz NMR equipped with an inverse 5 mm quadruple-resonance Z-grade cryoprobe (spectrometer frequencies: 700.43 MHz for ^1^H, 176.12 MHz for ^13^C). Prior to the measurements, the purified lipid X was exchanged twice from CDCl_3_/CD_3_OD 2:1 (v/v). Deuterated solvents were purchased from Deutero GmbH (Kastellaun, Germany). All data were acquired and processed using the Bruker TOPSPIN software (version 3.0 or higher). The parameter sets used were adapted from the respective Bruker standard parameter sets, which are all included in this software. Chemical shifts were referenced to internal chloroform (δ_H_ = 7.26 ppm, δ_C_ = 77.16 ppm) (Gottlieb et al. 1997). The ^1^H NMR assignments were confirmed by two-dimensional (2D) ^1^H,^1^H-COSY and total correlation spectroscopy (TOCSY) experiments. ^13^C NMR assignments were indicated by 2D ^1^H,^13^C-HSQC, based on the ^1^H NMR assignments. Inter-residue connectivity and further evidence for ^13^C assignment were obtained from 2D ^1^H,^13^C-heteronuclear multiple bond correlation and ^1^H,^13^C-HSQC-TOCSY experiments.

### Isolation and measurement of cuticular waxes and cutin monomers

*N. benthamiana* leaves were infiltrated with *Agrobacterium* cells harboring the PES2 construct. The infiltrated leaf areas were dipped in chloroform for 10 s to extract cuticular waxes without dissolving internal leaf lipids. The wax diester fraction was isolated by solid-phase extraction and analyzed by nanospray Q-TOF MS/MS as described above or by GC–MS (Baales et al. 2021).

For cutin analysis, leaf discs were dissected from the center of secondary leaves of wild-type *N. benthamiana* plants. The discs were cut into small pieces and all lipids were extracted with chloroform/methanol 1:1 (v/v) by shaking three times with fresh solvent each time for 4 h. Next, the leaf pieces were dried on a Teflon support overnight. The dried leaf material (3 mg) was incubated in 2 ml BF_3_/methanol at 70 °C for 16 h in an airtight vial for transesterification of the cutin polymer. After cooling to room temperature, the internal standard (10 µg dotriacontane) was added and the samples were transferred to vials containing 2 ml NaHCO_3_/H_2_O. The cutin monomers were extracted three times with 2 ml chloroform. The combined organic phases were washed with 2 ml water and dried with anhydrous Na_2_SO_4_. The sample volume was reduced to 200 μl by evaporation under an N_2_ stream, and 20 μl of pyridine and 20 μl of BSTFA were added for derivatization. After incubation at 70 °C for 40 min, the derivatized cutin monomers were quantified by GC with flame ionization detector (hydrogen served as the carrier gas; 2 ml min^−1^ flow rate) and identified by GC–MS (Agilent 5977 A series GC, MSD MS) on a DB-1 column (30 m length, 0.32 mm diameter, 0.1 µm film). After on-column injection of 1 µl of sample, the cutin monomers were eluted with the following temperature program (initial, 50 °C, 2 min; 10 °C min^−1^ to 150 °C; 150 °C, 1 min; 3 °C min^−1^ to 310 °C; 310 °C, 20 min).

## Results

### Fatty acid phytyl ester production after expression of *Arabidopsis* PES2 in *N. benthamiana* leaves

**Fig. 1:**
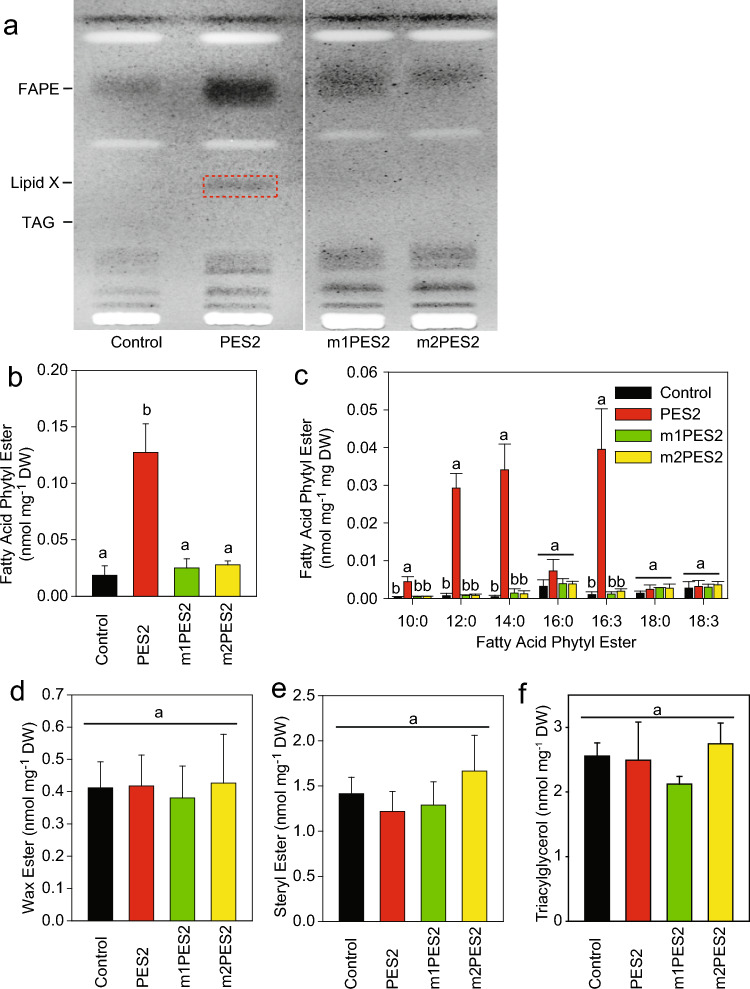
Accumulation of fatty acid phytyl esters (FAPEs) and of an unknown lipid X after expression of PES2 in *N. benthamiana*. *N. benthamiana* leaves were infiltrated with *Agrobacterium* cells containing different expression constructs (PES2, full length; m1PES2, m2PES2, truncated ORFs lacking the first 64 or 93 N-terminal amino acids, respectively). Lipids were isolated and purified by solid-phase extraction. **a** Neutral lipids were separated by TLC and visualized under UV light after primuline staining. Expression of PES2 resulted in the accumulation of fatty acid phytyl esters (FAPEs) and of lipid X (dashed red box), both absent in the control (transformed with empty vector); **b** total FAPE content; **c** acyl composition of FAPE; **d** wax esters; **e** steryl esters; **f** triacylglycerol (TAG). Lipids were measured by Q-TOF–MS/MS. Mean ± SD; n = 5. One-way ANOVA with post-hoc Tukey test. Letters indicate significant differences between the expression constructs. *P* < 0.05

Previously, co-expression of *Arabidopsis* PES2 with an acyl-CoA reductase (FAR) in *N. benthamiana* leaves had led to the accumulation of hexadecanol (16:0ol) or octadecanol (18:0ol) esters, depending on the origin of the FAR enzyme from *Arabidopsis* (AtFAR6) or *Marinobacter* (MaFAR), respectively. No wax esters were produced in experiments lacking FAR expression (Aslan et al. 2014). To study the specificity of *Arabidopsis* PES2 for endogenous alcohols, *N. benthamiana* leaves were infiltrated with *Agrobacterium* cells harboring the PES2 expression construct, but without AtFAR6 or MaFAR. Three different constructs were used, the full-length PES2 and two N-terminally truncated forms (m1PES2, m2PES2) lacking the predicted chloroplast targeting sequence (see Materials and methods). Four to seven days after infiltration, lipids were isolated from the leaves. Separation of lipids isolated from PES2-expressing *N. benthamiana* leaves by TLC revealed a band that co-migrated with FAPE and was identified as such by Q-TOF MS/MS. This band was strongly decreased in lipid extracts from leaves expressing m1PES2 or m2PES2 (Fig. 1a). The expression of PES2 resulted in a sevenfold increase in FAPE content compared to the control as measured by Q-TOF MS/MS (Fig. 1b). The FAPEs contained mainly 12:0, 14:0, and 16:3 fatty acids, and low amounts of 10:0, 16:0, 18:0, and 18:3 (Fig. 1c), similar to the FAPE pattern in chlorotic *Arabidopsis* leaves. The accumulation of medium-chain fatty acids and of 16:3 in FAPEs indicates that the acyl groups in PES2-expressing *N. benthamiana* leaves are presumably derived from the chloroplast fatty acid de novo synthesis and from MGDG, respectively (Lippold et al. 2012). In contrast, the expression of truncated PES2 constructs (m1PES2, m2PES2) did not result in increased FAPE production in *N. benthamiana* leaves as compared to the control, indicating that PES2 must be targeted to the chloroplast to produce FAPE.

To address the question of whether PES2 can produce other lipids in addition to FAPEs, wax esters, steryl esters, and triacylglycerol (TAG) were measured in PES2-expressing *N. benthamiana* leaves by Q-TOF MS/MS. The amounts of wax esters, steryl esters, or TAG did not increase after the expression of PES2, m1PES2, or m2PES2*,* presumably due to the limited availability of substrates (Fig. 1 d, e, f) (Aslan et al. 2014).

### Accumulation of hexadecanediol diesters after expression of *Arabidopsis* PES2 in *N. benthamiana*

**Fig. 2:**
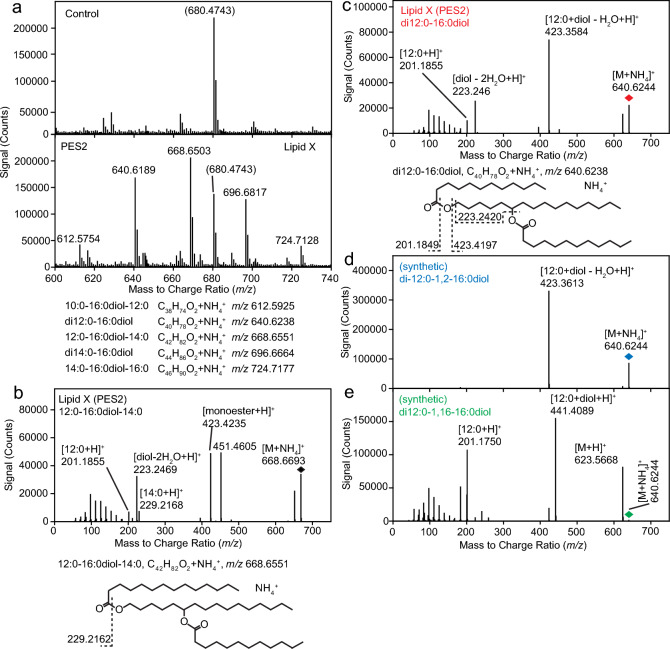
Identification of hexadecanediol diesters in *N. benthamiana* leaves expressing PES2. **a** Lipid X was isolated by TLC (Fig. 1) and analyzed by Q-TOF MS. The mass spectrum of lipid X shows a series of peaks with *m/z* values differing by ~ 28 Da corresponding to a C_2_H_4_ moiety, and these peaks were absent from the control. The calculated *m/z* values for the hexadecanediol diesters are shown below. The peak at *m/z* 680.4743 in the two spectra is an unknown contaminant, possibly corresponding to tris(2,4-di-*tert*-butylphenyl) phosphate, a polymer stabilizer with a calculated *m/z* of 680.4802 (Schuhmann et al. 2012). **b** Q-TOF MS/MS spectrum of *m/z* 668.6693 from lipid X which corresponds to 12:0–16:0diol-14:0. The neutral loss of 222.2399 and the fragment ion of *m/z* 223.2469 correspond to a hexadecanediol (C_16_H_34_O_2_). Fragmentation patterns are shown below. The linkage of 12:0 and 14:0 to the C_1_ or C_6_ hydroxyl groups is unknown. **c** Q-TOF MS/MS spectrum of *m/z* 640.6244 from lipid X corresponding to di12:0–16:0diol. Fragmentation patterns are shown below. **d** Q-TOF MS/MS spectrum of di12:0–1,2–16:0diol synthesized from 12:0 and 1,2–16:0diol and purified by TLC. **e** Q-TOF MS/MS spectrum of di12:0–1,16–16:0diol synthesized from 12:0 and 1,16–16:0diol and purified by TLC. The MS/MS spectra of the molecular ions *m/z* 640.6244 (diamonds) from lipid X (red), synthetic di12:0–1,2–16:0diol (blue), and synthetic di12:0–1,16–16:0diol diester (green) reveal different fragmentation patterns

An additional band (lipid X) was observed on the TLC plate, which migrated between FAPE and TAG (Fig. 1a, dashed box). This novel lipid X accumulated in leaves expressing PES2, but it was absent from control leaves or from leaves expressing m1PES2 or m2PES2. Therefore, the accumulation of lipid X was dependent on the targeting of PES2 to the chloroplasts. The amount of lipid X produced in PES2-expressing leaves was much lower compared with FAPE, as judged by the stained lipid bands. Lipid X was isolated from the TLC plate and subjected to highly sensitive Q-TOF MS analysis. The total ion spectrum revealed a series of peaks with masses ranging from *m/z* 612.5754 to 724.7128 (Fig. 2a). The difference between two consecutive masses was ~ 28, corresponding to a C_2_H_4_ moiety indicative for lipids containing aliphatic chains. The fragmentation pattern of the *m/z* 668.6503 peak revealed fragments at *m/z* 451.4605 [14:0 + hexadecanediol-H_2_O + H]^+^, 423.4235 [12:0 + hexadecanediol-H_2_O + H]^+^, 229.2168 [14:0 + H]^+^, and 201.1855 [12:0 + H]^+^, with neutral losses of 217.2043 [12:0 + NH_3_] and 245.2458 [14:0 + NH_3_] (Fig. 2b). The neutral loss of 222.2380 and the fragment of 223.2469 correspond to a hexadecanediol (C_16_H_30_) lacking two water molecules. Thus, *m/z* 668.6503 was identified as a wax diester of hexadecanediol with 12:0 and 14:0 fatty acids. Fragmentation of the *m/z* 640.6189 ion resulted in three fragments at *m/z* 423.3584 [12:0 + hexadecanediol–H_2_O + H]^+^, 223.2461 [C_16_H_30_ + H]^+^ and 201.1855 [12:0 + H]^+^, with successive neutral losses of 222.2427 [C_16_H_30_], 217.2081 [12:0 + NH_3_] and 200.1821 [12:0] (Fig. 2c). Therefore, the *m/z* 640.6244 ion was identified as a hexadecanediol diester with two 12:0 fatty acids. Analysis of other parental ions demonstrated that the series of lipids produced by PES2 in *N. benthamiana* leaves consists of hexadecanediol diesters with different saturated fatty acids.

For the identification of the hydroxyl positions in hexadecanediol, first commercially available 1,2-hexadecanediol (1,2–16:0diol) and 1,16-hexadecanediol (1,16–16:0diol) standards were used to chemically synthesize wax diesters with 12:0 fatty acid. The fragmentation patterns of lipid X from PES2-expressing leaves and synthetic di12:0–1,2-hexadecanediol and di12:0–1,16-hexadecanediol were compared by Q-TOF MS/MS (Fig. 2d, e). All three hexadecanediol diesters ionized as ammonium adducts [M + NH_4_]^+^ of *m/z* 640.6244. In contrast to the spectrum of *m/z* 640.6244 from lipid X, only the 12:0-monoester *m/z* 423.3613, but no other product ions were found for di12:0–1,2-hexadecanediol (Fig. 2d). The fragmentation of di12:0–1,16-hexadecanediol [M + NH_4_]^+^ was also different, showing two neutral losses of the NH_3_ group resulting in the [M + H^+^] ion at *m/z* 623.5668, and of [12:0–H_2_O] resulting in the monoester [12:0 + 16:0diol + H]^+^ at *m/z* 441.4089. In addition, an acyl peak [12:0 + H]^+^ was found at *m/z* 201.1750, while the hexadecanediol peak (*m/z* 223.2426) was barely visible (Fig. 2e). Since the fragmentation patterns of lipid X and the two synthetic hexadecanediol diesters were different, the hexadecanediol in lipid X must have a different structure than 1,2-hexadecanediol or 1,16-hexadecanediol.

### The wax diesters produced by PES2 contain a unique 1,6-hexadecanediol

**Fig. 3:**
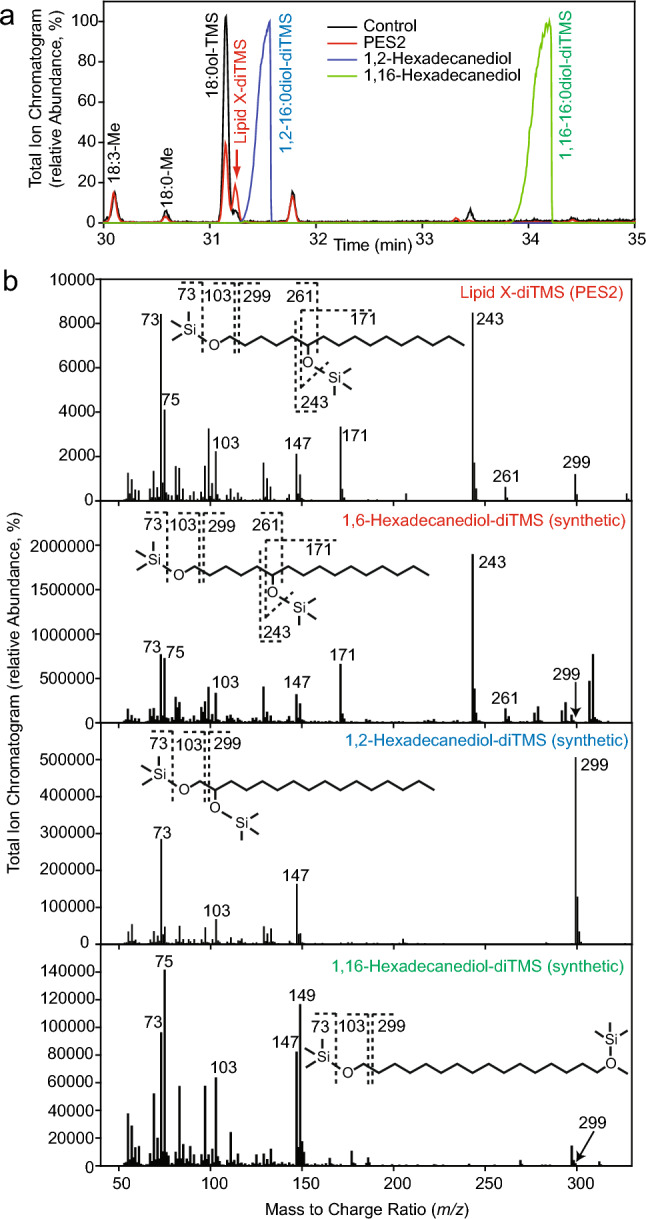
GC–MS analysis of the hexadecanediol obtained after hydrolysis of lipid X from *N. benthamiana* leaves expressing PES2. **a** Wax diesters were cleaved with methanolic NaOH; the hexadecanediols were converted into trimethylsilyl (TMS) ethers and separated by GC–MS. Black, chromatogram of *N. benthamiana* control leaves expressing GFP; red, *N. benthamiana* leaves expressing PES2 (lipid X); blue, 1,2-hexadecanediol-diTMS standard; green, 1,16-hexadecanediol-diTMS standard. 18:0-Me, 18:3-Me, and 18:0ol-TMS correspond to fatty acid methyl esters or to a trimethylsilyl derivative of 18:0ol, respectively. **b** GC–MS spectra of the hexadecanediol-diTMS obtained from hydrolysis of lipid X, and of synthetic standards (red, 1,6-hexadecanediol-diTMS; blue, 1,2-hexadecanediol-diTMS; green, 1,16-hexadecanediol-diTMS

Next, lipid X isolated from PES2 expressing leaves was cleaved by acidic hydrolysis and the hydrolyzed hexadecanediol was converted to trimethylsilyl ethers (TMS) prior to analysis by GC–MS. The results were compared with those obtained from leaves infiltrated with empty vector and with the standards 1,2–16:0diol, 1,16–16:0diol, and synthetic 1,6–16:0diol. The chromatogram of the hexadecanediol derived from lipid X displayed a peak (lipid X-diTMS) at 31.25 min which was absent from the chromatograms of the control and the 1,2–16:0diol-diTMS and 1,16–16:0diol-diTMS standards (Fig. 3a, red arrow). The spectrum of lipid X-diTMS showed ions with *m/z* 73 (Me_3_Si^+^) (Me, methyl), *m/z* 75 (Me_2_Si-OH^+^), and *m/z* 147 (Me_3_Si-O^+^ = SiMe_2_) derived from TMS groups, indicative for an alkanediol (Fig. 3b) (Richter and Burlingame 1968; Jetter et al. 1996). The fragment *m/z* 103 is indicative for a primary hydroxyl group at C1. The ion *m/z* 243 corresponding to [C_11_H_22_O-SiMe_3_]^+^ is derived from the α-cleavage of an alkanediol with a secondary hydroxyl group at C6 (Jetter et al. 1996). The GC–MS spectrum was very similar to that of synthetic 1,6-hexadecanediol-diTMS, but different from that of 1,2-hexadecanediol-diTMS and 1,16-hexadecanediol-diTMS (Fig. 3b). Therefore, the GC–MS spectrum of the lipid X peak at 31.25 min corresponds to 1,6-hexadecanediol-diTMS.

The identity of the hexadecanediol derived from lipid X was confirmed by LC–MS. To this end, lipid X from PES2-expressing leaves was cleaved by acidic hydrolysis and its retention time during LC–MS was compared with the 1,16-hexadecanediol and 1,6-hexadecanediol standards (Fig. S1a). The hexadecanediol of lipid X eluted at 15.61 min, after 1,16-hexadecanediol (15.26 min), and it co-eluted with 1,6-hexadecanediol (15.65 min), indicating that lipid X contains 1,6-hexadecanediol. To address the question of whether additional alkanediols are present in lipid X, MS/MS/MS experiments were performed after in-source fragmentation with increased fragmentor voltage (Fig. S1b). Each monoester ion [fatty acid + hexadecanediol–H_2_O + H]^+^ was selected in the quadrupole and fragmented again, resulting in the occurrence of only one specific fatty acid adduct [fatty acid + H]^+^. Therefore, the series of wax diesters of lipid X contains only 1,6-hexadecanediol.

^1^H-NMR and ^13^C-NMR spectra were finally recorded to confirm the structure of the hexadecanediol diester derived from lipid X. To this end, lipid X from ~ 150 infiltrated *N. benthamiana* leaves expressing PES2 was isolated and purified by solid-phase extraction and TLC. The ^1^H-NMR and ^13^C-NMR analyses showed characteristic shifts for 1-H, 6-H, and the C1, C6, respectively, conclusively demonstrating that the alkanediol in the wax diesters is 1,6-hexadecanediol (Fig. S2, Table S4).

### Wax diesters produced by PES2 are rich in medium-chain fatty acids and localize to the chloroplasts

**Fig. 4:**
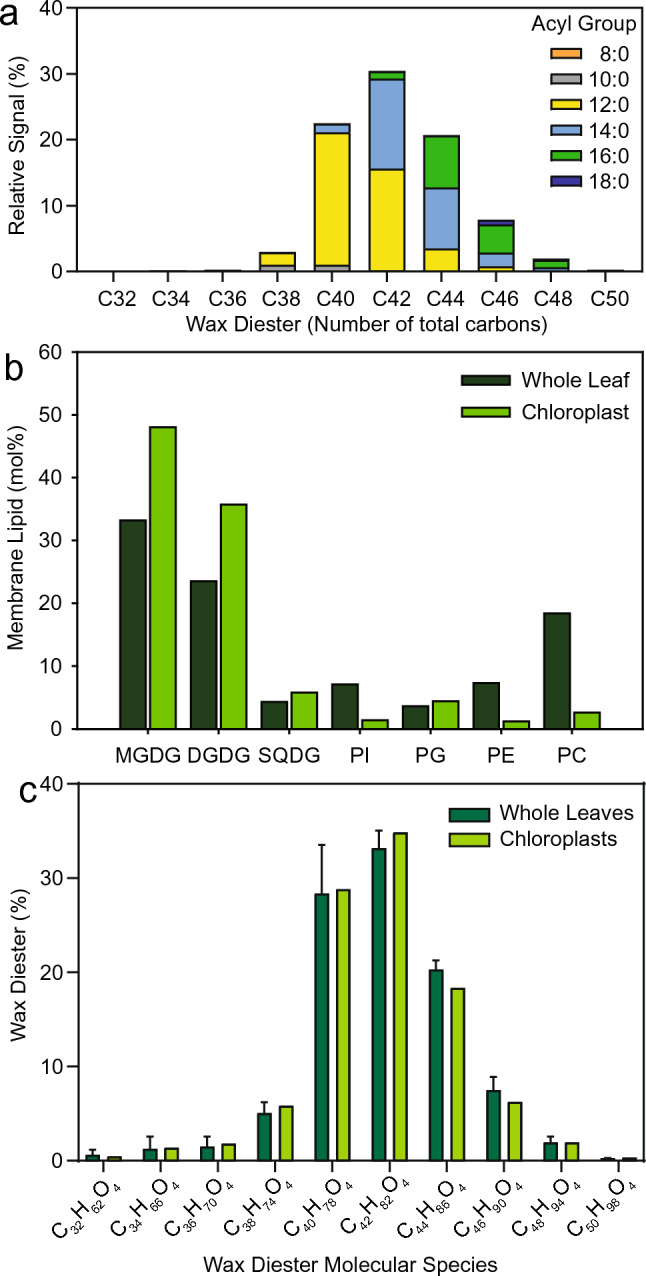
Fatty acid composition and subcellular localization of 1,6-hexadecanediol diesters. **a** Fatty acid distribution in 1,6-hexadecanediol diesters produced by PES2 in *N. benthamiana*. 1,6-Hexadecanediol diesters were isolated from *N. benthamiana* leaves expressing PES2, purified by solid-phase extraction, and measured by neutral loss scanning of [fatty acid + NH_3_] using Q-TOF MS/MS. Means; n = 2. **b** Membrane lipid composition of whole leaves and chloroplasts measured by direct infusion Q-TOF MS/MS. Chloroplasts are rich in MGDG, DGDG, SQDG, and PG, but contain only low amounts of the outer chloroplast envelope and extraplastidial lipids PC, PI, and PE. **c** Accumulation of hexadecanediol diesters in chloroplasts of *N. benthamiana* leaves expressing PES2. Hexadecanediol diesters were isolated from whole leaves and from chloroplasts and measured by direct infusion MS/MS. Means ± SD; n = 2 for whole leaves; one representative result for isolated chloroplasts. For the molecular species of wax diesters, see Table S3

Since all wax diesters produced by PES2 contain 1,6-hexadecanediol, the ions observed by mass spectrometry must contain different acyl groups. Neutral loss scanning was used to study the composition of fatty acids bound in the wax diesters. The three most abundant wax diesters contain a total of 40, 42, or 44 carbon atoms (Fig. 4a). The most abundant fatty acids are 12:0, followed by 14:0 and 16:0. No unsaturated fatty acids were found. Hexadecatrienoic acid (16:3) which is highly abundant in FAPE produced in PES2-expressing *N. benthamiana* leaves was not detected in the wax diesters.

To study the subcellular accumulation of wax diesters, chloroplasts of PES2-expressing *N. benthamiana* leaves were isolated. Lipids were extracted from the isolated chloroplasts and from whole leaves. The purity of isolated chloroplasts can be assessed by membrane lipid measurements, because the chloroplast lipids are enriched in MGDG, DGDG, SQDG, and PG, but contain minor amounts of the non-chloroplast lipids PE, PI, and PC which are found in extraplastidial membranes and the outer chloroplast envelope. The quantification of membrane lipids from isolated chloroplasts confirmed their purity, as evidenced by the low abundance of PE, PI, and PC in comparison to whole leaf extracts (Fig. 4b). Wax diesters were purified by solid-phase extraction and quantified by Q-TOF MS/MS (Fig. 4c). Hexadecanediol diesters were identified in the chloroplast sample with a molecular species distribution very similar to that of whole leaves. Therefore, hexadecanediol diesters produced upon PES2 expression accumulate in the chloroplasts of *N. benthamiana*. The acylation of hexadecanediol by PES2 presumably traps 1,6-hexadecanediol in the chloroplasts, as it might otherwise be exported to the cytosol in its free form to serve as an intermediate in an unknown biosynthetic pathway.

### Hexadecanediol and hexadecanediol diesters are absent from the wax and cutin layers of *N. benthamiana* leaves

**Fig. 5:**
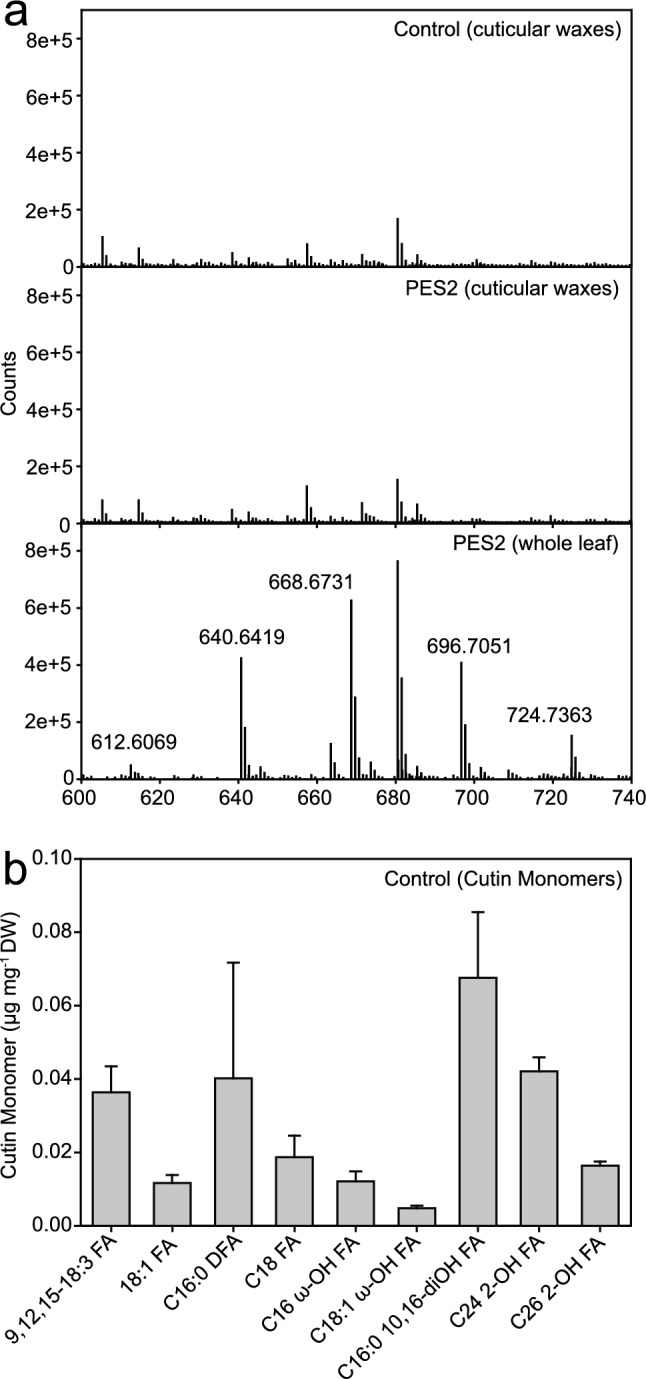
Localization of 1,6-hexadecanediol diesters in leaves of *N. benthamiana* expressing PES2. **a** Analysis of cuticular waxes by direct infusion Q-TOF MS/MS. Cuticular waxes were isolated from control leaves (top) or leaves infiltrated with *Agrobacterium* cells harboring the PES2 construct (middle) by dipping in chloroform. The bottom panel shows lipids isolated from whole leaves expressing PES2. For the *m/z* values of hexadecanediol diesters, see Fig. 2. **b** Identification and quantification of cutin monomers from *N. benthamiana* leaves. Leaf discs of *N. benthamiana* wild-type control plants were dilapidated and the remaining cutin polymer was transesterified. The monomers were silylated, and the compounds were identified by GC–MS and quantified by GC-FID. Means ± SD; n = 3. 9,12,15–18:3 FA, α-linolenic acid; 18:1 FA, oleic acid; C16:0 DFA, hexadecanedioic acid; C18 FA, stearic acid; C16 ω-OH FA, ω-hydroxypalmitic acid; C18:1 ω-OH FA, ω-hydroxyoleic acid; C16:0 10,16-diOH FA, 10,16-dihydroxypalmitic acid; C24 2-OH FA, 2-hydroxytetracosanoic acid; C26 2-OH FA, 2-hydroxyhexacosanoic acid

Figure 1 shows that the wax diesters of 1,6-hexadecanediol are only produced when PES2 is targeted to the chloroplasts. Alkanediols are usually found in plant cuticular waxes or in the suberin layer, while their synthesis takes place at the ER (Jetter et al. 2006; Li-Beisson et al. 2013). To study whether the PES2-dependent wax diesters are transported to the leaf surface and deposited in the cuticular wax layer, surface waxes of control and PES2-infiltrated *N. benthamiana* leaves were obtained by dipping the leaf in chloroform. GC–MS analysis revealed the presence of C16, C18, C20 fatty acids, C27, C29, C31, C33 alkanes, and C18, C20, C22, C24, C26 alcohols, as previously described for *N. benthamiana* waxes (Asadyar et al. 2024). Hexadecanediol or hexadecanediol diesters were not detected. Then, we used highly sensitive Q-TOF MS/MS analysis and confirmed that surface waxes of control and PES2-expressing leaves were devoid of hexadecanediol diesters, in contrast to whole PES-expressing leaves, indicating that hexadecanediol diesters produced in the chloroplast by PES2 are not transported to the cuticle wax layer (Fig. 5a).

Next we investigated whether 1,6-hexadecanediol accumulates in the cutin layer of *N. benthamiana* leaves. The soluble wax lipids were extracted and the cutin polymer was depolymerized. Analysis of the cutin monomers revealed the presence of free fatty acids, dicarboxylic acids, and hydroxy fatty acids, consistent with previous studies (Fawke et al. 2019). Alkanediols were not detected (Fig. 5b). Therefore, hexadecanediol produced in the chloroplast is not incorporated into the cutin layer on the leaf surface.

## Discussion

### PES2 harbors acyltransferase activity with broad specificity for different alcohols

PES2 belongs to the ELT family of acyltransferases which has six members in *Arabidopsis*. Together with PES1, PES2 was originally identified as a chloroplast-localized FAPE synthase in *Arabidopsis* (Lippold et al. 2012). Expression of PES2 in *N. benthamiana* leaves resulted in the accumulation of wax esters, but only after co-transformation with an acyl-CoA reductase (FAR) (Aslan et al. 2014). Expression of PES2 without FAR in *N. benthamiana* resulted in the accumulation of FAPE, but no wax esters, steryl esters, or TAG were produced, presumably because the corresponding substrates (sterols, long-chain alcohols, diacylglycerols) are limiting in the chloroplasts (Fig. 1). However, wax diesters of 1,6-hexadecanediol were produced by PES2 in chloroplasts of *N. benthamiana* leaves, indicating that PES2 harbors wax diester synthase activity. Confirmation of the wax diester synthase activity (i.e., acylation of positions 1 and 6 of 1,6-hexadecanediol) by feeding or in vitro enzyme assays is challenging due to the low activity of recombinant PES2 and the limited supply of the substrate 1,6-hexadecanediol which is not commercially available. PES2 has a broad substrate specificity, as it can acylate phytol, diacylglycerol, and xanthophylls (Fu et al. 2025, Lippold et al. 2012). PES1 and PES2 were found to be required for the accumulation not only of xanthophyll mono- and diesters but also of neoxanthin triesters in *Arabidopsis* callus, indicating that they acylate up to three hydroxyl groups on the two rings of neoxanthin (Fu et al. 2025). In *Synechocystis*, the PES2-related slr2103 acyltransferase can produce triacylglycerol, FAPEs, and it acylates the quinol and side-chain hydroxyl groups of plastoquinol and plastoquinol C (Shajil Das et al. 2025). It is therefore highly probable that PES2 acylates both positions of 1,6-hexadecanediol.

### Identification of 1,6-hexadecanediol diesters in *N. benthamiana* leaves expressing *Arabidopsis* PES2

Analysis of non-polar lipids of PES2-expressing *N. benthamiana* leaves by TLC revealed the existence of an additional lipid X that was identified as 1,6-hexadecanediol diesters with saturated fatty acids ranging from 10:0 to 16:0, with 12:0 and 14:0 being the most abundant. This fatty acid composition correlates with the fatty acid preference of PES2 during FAPE synthesis in *Arabidopsis*, although no 16:3 was incorporated into 1,6-hexadecanediol diesters in *N. benthamiana*. Thus, PES2 esterifies only saturated fatty acids to 1,6-hexadecanediol, while 16:3 and saturated fatty acids are employed for FAPE synthesis. This specificity might be related to the structure of the acyl donor, as 16:3 could be derived from 16:3-containing MGDG, whereas 10:0, 12:0, and 14:0 presumably originate from the acyl-ACP pool (Lippold et al. 2012).

Previously, alkanediol diesters or free alkanediols were found as constituents of the plant cuticle (Jetter et al. 2006). Alkanediol diesters have been identified in cuticular waxes of wheat, oat, and rye (Tulloch 1971; Tulloch and Hoffman 1974). Non-esterified alkanediols have also been found in the cuticular waxes of pine tree needles (*Pinus radiata)* (Franich et al. 1979), ferns fronds (*Osmunda regalis*) (Jetter and Riederer 1999), and in leaves of *Myricaria germanica* (Jetter 2000), pea (*Pisum sativum)* (Wen et al. 2006), and poppy (*Papaver)* (Jetter et al. 1996). *Arabidopsis* stems contain free alkanediols in the cuticular wax layer (Wen and Jetter 2009), and alkanediols were found as monomers of the suberin polyester of the seeds (Molina et al. 2006). However, no fatty alcohols or alkanediols were found in the cuticular waxes or cutin polymers of leaves of different *Nicotiana* species (Heemann et al. 1983; Fawke et al. 2019). Therefore, 1,6-hexadecanediol reported here is not a component of the cuticular waxes or the cuticle of *N. benthamiana*.

### 1,6-Hexadecanediol and its diesters are synthesized in chloroplasts of *N. benthamiana* leaves

Precursors of cuticular waxes and monomers of the cutin polymer are believed to be synthesized exclusively in the ER of epidermal cells and transported to the leaf surface (Li-Beisson et al. 2013). After infiltration into *N. benthamiana* leaves, hexadecanediol diesters were synthesized only when PES2 was expressed as a full-length protein containing the predicted N-terminal chloroplast targeting sequence, indicating that hexadecanediol diesters are produced in the chloroplast. It is possible that some 1,6-hexadecanediol or diesters are exported from chloroplasts, possibly to the ER. Infiltration of *N. benthamiana* leaves with *Agrobacterium* cells carrying a construct with the CaMV 35S promoter results in expression in mesophyll and epidermal cells (Juneidi et al. 2014). However, leaf surface waxes isolated after dipping of PES2-expressing *N. benthamiana* leaves in chloroform and the cutin monomer fraction were devoid of hexadecanediol or hexadecanediol diesters. Therefore, it is likely that hexadecanediol diesters accumulate in the chloroplasts of the mesophyll cells in transformed leaves. Non-polar lipids, including FAPE, fatty acid xanthophyll esters, and tocopherols, are stored in the plastoglobules of chloroplasts. In addition, PES2 has been found in the plastoglobule proteome (Vidi et al. 2006; Ytterberg et al. 2006). Therefore, it is likely that hexadecanediol diesters are produced by PES2 in the plastoglobules.

### A possible pathway for the synthesis of 1,6-hexadecanediol in *N. benthamiana*

**Fig. 6:**
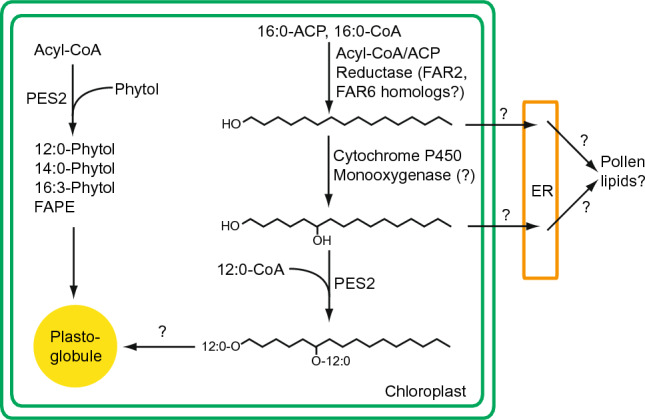
Putative pathway for FAPE and 1,6-hexadecanediol diester synthesis in *N. benthamiana* leaves expressing *Arabidopsis* PES2. Infiltration of *N. benthamiana* leaves with PES2 results in the accumulation of FAPE and 1,6-hexadecanediol diesters. FAPE is synthesized in the chloroplasts from acyl-CoA and free phytol. Hexadecanol can be synthesized by chloroplast-localized acyl-CoA or acyl-ACP reductases, orthologs of *Arabidopsis* FAR2 or FAR6. The second hydroxyl group at position 6 is presumably introduced by a cytochrome P450 monooxygenase. Esterification with medium-chain fatty acids is catalyzed by *Arabidopsis* PES2. FAPE and 1,6-hexadecanediol diesters are synthesized in the chloroplasts and presumably localized to the plastoglobules. In anthers, 1,6-hexadecanediol diesters might be exported from the chloroplasts to the ER for pollen lipid production

Primary alcohols, the putative precursors for the synthesis of alkanediols, are components of the cuticular waxes and monomers of cutin polymers. The alcohols are synthesized via reduction of acyl-CoA or acyl-ACP by FARs. While the FAR enzymes from *N. benthamiana* have not been studied, it has been shown that *Arabidopsis* contains a family of eight FARs, six of which are localized to the ER (Rowland and Domergue 2012), while FAR2 (synonymous MS2) and FAR6 are localized to the chloroplasts and show substrate specificity for palmitoyl-ACP (16:0-ACP) (Chen et al. 2011; Doan et al. 2012). Thus, the action of the two plastidial FARs or their orthologs from *N. benthamiana* leads to the production of 1-hexadecanol in the chloroplasts (Fig. 6). Secondary alcohols can be synthesized by hydroxylases via mid-chain hydroxylation of alkanes or primary alcohols. In *Arabidopsis*, the mid-chain alkane hydroxylase 1 (MAH1) is involved in the production of secondary alcohols as components of the stem cuticular waxes (Greer et al. 2007; Wen and Jetter 2009). MAH1 is a member of the large family of cytochrome P450 enzymes (CYP), which contains 272 proteins in *Arabidopsis* (Bak et al. 2011). The in-chain hydroxylation of 1-hexadecanol yielding 1,6-hexadecanediol could be catalyzed by a chloroplast-localized ortholog of MAH1 in *N. benthamiana*. Our attempts to identify 1,6-hexadecanediol in plants other than *N. benthamiana*, including *Arabidopsis,* were unsuccessful. Therefore, the pathway of plastidial alkanediol synthesis might be restricted to some plants such as *N. benthamiana*. A major difference between MAH1 and the putative secondary alcohol producing activity of *N. benthamiana* is the regiospecificity, as MAH1 catalyzes the single or multiple hydroxylation of vicinal carbons located near the center of the aliphatic chain (of C27 to C31 alcohols) leading to hydroxyl groups between C13 and C15 (Wen and Jetter 2009). However, the in-chain hydroxylation in *N. benthamiana* chloroplasts is exclusively localized at position 6, resulting in the production of 1,6-hexadecanediol.

An alternative pathway for the introduction of in-chain hydroxyl groups has previously been proposed (Wettstein-Knowles 1995). Fatty acid synthesis in the chloroplast or at the ER starts with the addition of a C2 unit from malonyl-ACP or malonyl-CoA, introducing a C3 keto function that is subsequently reduced to a hydroxyl group. If this hydroxyl group is not further dehydrated, it will remain in the acyl chain, and after further elongation reactions, can give rise to a hydroxyl group at position 3, 5, 7, etc. However, it is difficult to imagine how this pathway could lead to the production of 1,6-hexadecanediol.

### Enzymes with wax diester synthase activity

To date, enzymes with wax diester synthase activity have only been characterized in non-plant species. The bifunctional wax ester synthase/acyl-CoA:diacylglycerol acyltransferase ADP1 from *Acinetobacter calcoaceticus* was heterologously expressed in *E. coli*. Upon feeding the cells with 1,16-hexadecanediol, wax diesters accumulated (Kalscheuer and Steinbüchel 2003). In addition, feeding of *A. calcoaceticus* cells with 1,16-hexadecanediol resulted in the accumulation of wax diesters with a fatty acid composition reflecting the fatty acid profile of whole cells. Mutant mice deficient in the acyl-CoA:diacylglycerol acyltransferase DGAT1 gene showed atrophic sebaceous glands and abnormal fur with loss of wax diesters which are the major surface lipids on mouse skin (Smith et al. 2000). After heterologous expression in insect cells, DGAT1 revealed wax diester synthase activity in the presence of added 1,2-hexadecanediol (Yen et al. 2005). Four DGAT2-related wax ester synthases (WS) have been identified in the protozoon *Tetrahymena thermophila* (Biester et al. 2012). After heterologous expression in yeast, only TtWS2 revealed an additional wax diester synthase activity with two substrates, 1,2-dodecanediol or 1,12-dodecanediol.

### Possible functions of 1,6-hexadecanediol and its diesters in *N. benthamiana*

*N. benthamiana* contains five sequences (Nbe.v1.1.chr19g32400, Nbe.v1.1.chr09g38130, Nbe.v1.1.chr11g12680, Nbe.v1.1.chr01g31300, Nbe.v1.1.chr01g31310) related to the ELT/PES family proteins from *Arabidopsis* (tBLASTn search with the PES2/At3g26840 amino acid sequence against the *N. benthamiana* genome at https://nbenthamiana.jp/). Therefore, *N. benthamiana* likely contains FAPE synthases capable of acylating phytol, and possibly also of 1,6-hexadecanediol. However, when we tested for the presence of wax diesters in wild-type *N. benthamiana* leaves under senescence or nitrogen deprivation, when PES2 expression is induced and FAPE accumulate in *Arabidopsis*, no wax diesters were detected. Attempts to detect 1,6-hexadecanediol by GC–MS in leaves of *N. benthamiana* control plants were unsuccessful. Therefore, the activity of endogenous PES enzymes, even during senescence or nitrogen deprivation, is too low to produce wax diesters, compared with the strong overexpression of *Arabidopsis* PES2 in *N. benthamiana*, indicating that the 1,6-hexadecanediol pool in *N. benthamiana* is very low. 1,6-Hexadecanediol might be a biosynthetic intermediate that is rapidly converted into an unknown downstream product. Overexpression of PES2 presumably traps the 1,6-hexadecanediol intermediate by sequestration into the wax diester pool.

The function of 1,6-hexadecanediol in *N. benthamiana* chloroplasts remains enigmatic. It has been suggested that monohydric alcohols such as hexadecanol, produced in *Arabidopsis* chloroplasts by FAR6 or FAR2/MS2 (male sterile 2), are important for pollen exine production (Rowland et al. 2006; Chen et al. 2011). Similarly, DPW (defective pollen wall) and MS25 (male sterile 25) encode plastid-localized FARs involved in pollen exine formation and pollen maturation in rice and maize, respectively (Shi et al. 2011; Zhang et al. 2021). Therefore, it is possible that 1,6-hexadecanediol is not a leaf metabolite per se*,* but might play a role as an intermediate in the synthesis of protective lipids in other plant organs such as pollen. In this scenario, the accumulation of 1,6-hexadecanediol diesters in *N. benthamiana* leaves expressing PES2 could represent the trapping of 1,6-hexadecanediol, which is usually not found in leaves, but which could be an intermediate of lipid produced in other plant organs such as anthers.

## Supplementary Information

Below is the link to the electronic supplementary material.

Supplementary file1 (PDF 750 KB)

Supplementary file2 (DOCX 26 KB)

## Data Availability

All data generated or analyzed during this study are included in this published article and its supplementary information files.

## Abbreviations

ELT: Esterase/lipase/thioesterase
FAPE: Fatty acid phytyl ester
GC–MS: Gas chromatography-mass spectrometry
GFP: Green fluorescent protein
LC–MS: Liquid chromatography-mass spectrometry
PES: Phytyl ester synthase
TLC: Thin-layer chromatography
X, Y: Fatty acids or alcohols are abbreviated as X:Y or X:Yol, respectively, with X indicating the number of carbon atoms, and Y the number of double bonds
