# Supplementary material for: Identification of 1,6-hexadecanediol and its wax diesters in chloroplasts of Nicotiana benthamiana
Source: Planta. 2025 Oct 9;262(5):117. doi: 10.1007/s00425-025-04833-8 (PMC12511139; doi:10.1007/s00425-025-04833-8)
Supplement: Supplementary file 1 — Supplementary file1 (PDF 750 KB) [file 425_2025_4833_MOESM1_ESM.pdf]

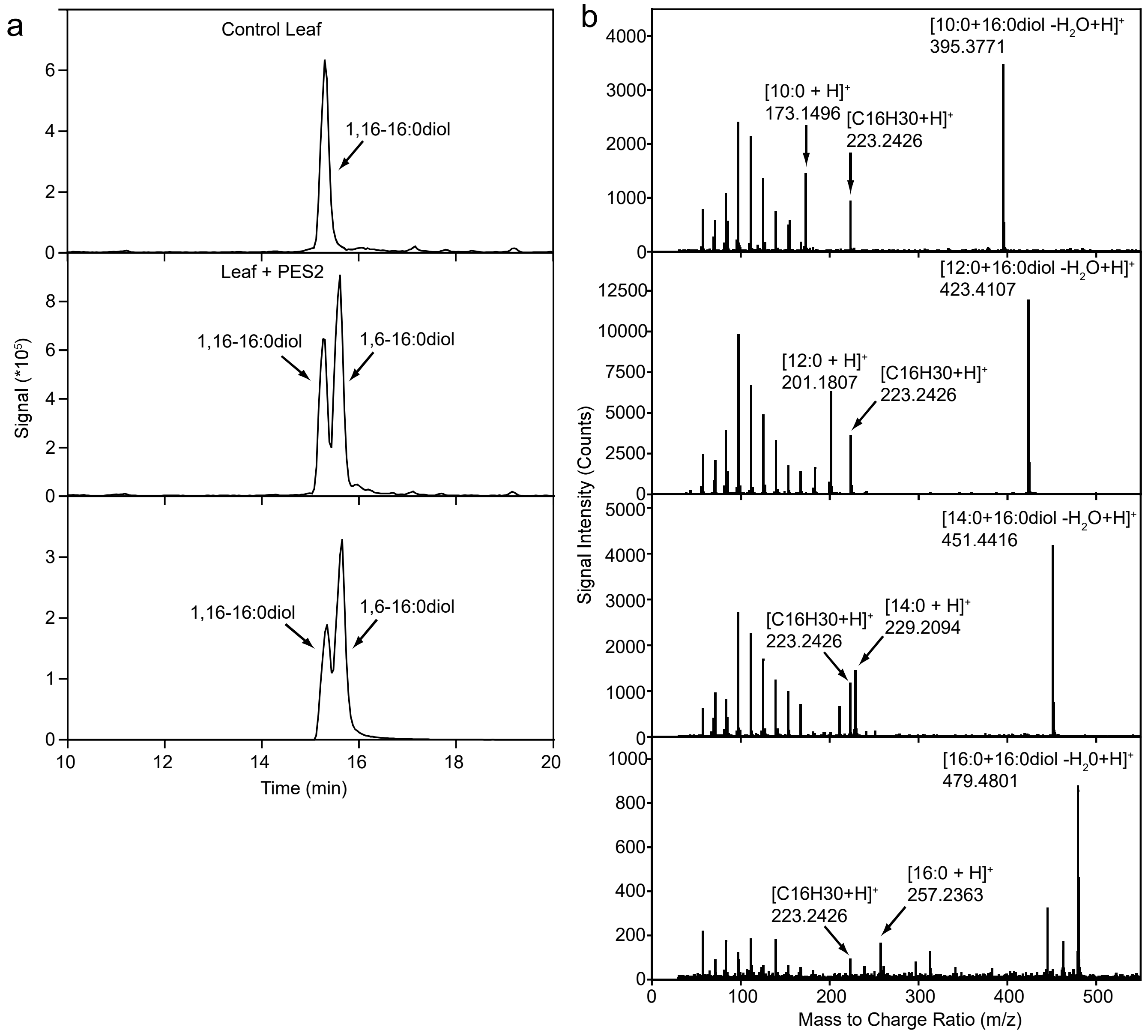

Fig. S1 Analysis of lipid X by LC-MS and in-source fragmentation.
a Lipids isolated by solid phase extraction from control leaves were cleaved by acidic hydrolysis and, together with the standard 1,16-16:0diol, separated via LC-MS by recording the parental ions $[M\text{-}H_2O+H]^+$ of m/z 241.2526 (top chromatogram). The chromatogram in the middle shows hydrolyzed lipids from PES2-expressing leaves with the standard 1,16-16:0diol. The bottom chromatogram shows the mixture of the two standards 1,16-16:0diol and 1,6-16:0diol. **b** In-source fragmentation was employed to generate monoester ions $[\text{fatty acid+diol–}H_2O+H]^+$ which were fragmented again in the quadrupole, resulting in the generation of a fatty acid ion $[\text{fatty acid}+H]^+$ and a di-dehydrated hexadecanediol ion at *m/z* 223.2426 $[C_{16}H_{30}+H]^+$. No other alkanediol peak was detected indicating that lipid X only contains 1,6-hexadecanediol.

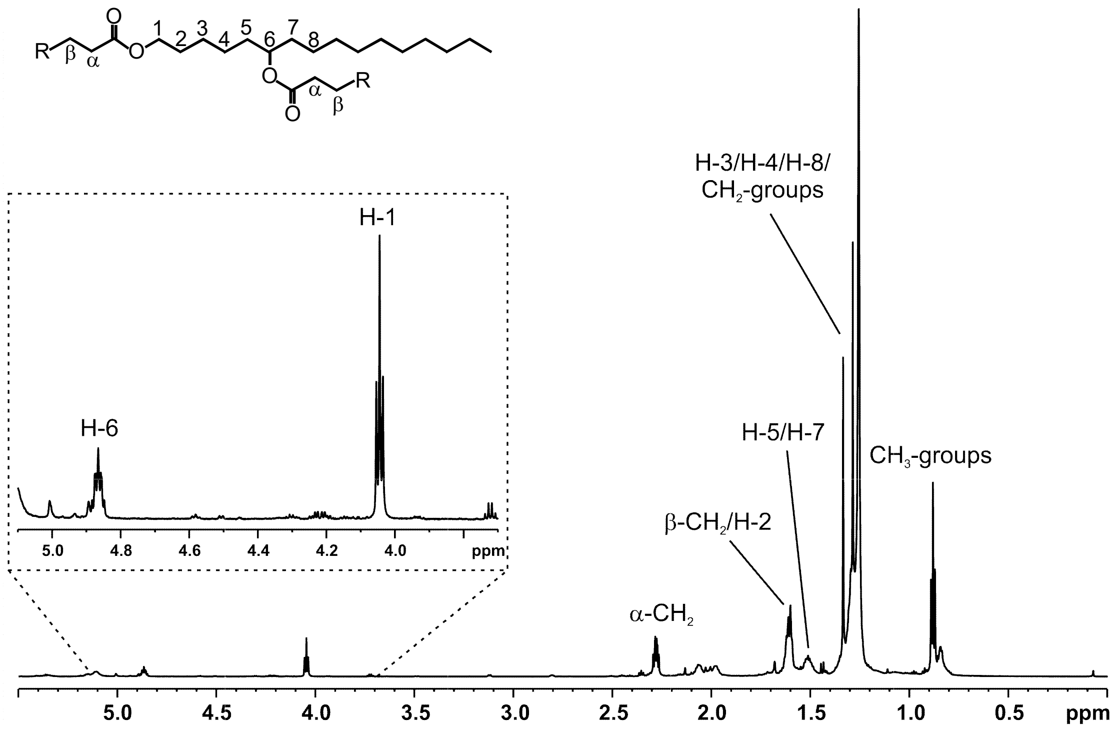

Fig. S2 Analysis of lipid X by NMR spectroscopy confirms that the alkanediol of the wax diesters is 1,6-hexadecanediol. Shown is the ^{1}H NMR spectrum (δ_H 5.5–0.0) of TLC-purified lipid X recorded in $CDCl_3$ at 300 K. The inset shows a close-up of the region between 5.1 and 3.7 ppm. For chemical shift assignments and coupling constants, see Table S4.
