# Supplementary material for: Identification of 1,6-hexadecanediol and its wax diesters in chloroplasts of Nicotiana benthamiana
Source: Planta. 2025 Oct 9;262(5):117. doi: 10.1007/s00425-025-04833-8 (PMC12511139; doi:10.1007/s00425-025-04833-8)
Supplement: Supplementary file 2 — Supplementary file2 (DOCX 26 KB) [file 425_2025_4833_MOESM2_ESM.docx]

**Table S1** Target list for Q-TOF MS/MS analysis of fatty acid phytyl esters (FAPE).

FAPE form ammonium adducts [M + NH_4_]^+^  in the positive mode and were measured by neutral loss scanning of the [phytol-H_2_O] fragment (278.2974). The collision energy was 5 V.

| Fatty Acid Phytyl Ester | Formula [M] | [M + NH_4_]^+^  (*m/z*) | Neutral Loss [phytol – H_2_O] |
| --- | --- | --- | --- |
| 8:0-phytol | C_28_H_54_O_2_ | 440.4468 | 278.2974 |
| 10:0-phytol | C_30_H_58_O_2_ | 468.4781 | 278.2974 |
| 12:0-phytol | C_32_H_62_O_2_ | 496.5094 | 278.2974 |
| 14:0-phytol | C_34_H_66_O_2_ | 524.5407 | 278.2974 |
| 16:3-phytol | C_36_H_64_O_2_ | 546.5250 | 278.2974 |
| 16:2-phytol | C_36_H_66_O_2_ | 548.5407 | 278.2974 |
| 16:1-phytol | C_36_H_68_O_2_ | 550.5563 | 278.2974 |
| 16:0-phytol | C_36_H_70_O_2_ | 552.5720 | 278.2974 |
| 17:0-phytol (I.S.) | C_37_H_72_O_2_ | 566.5876 | 278.2974 |
| 18:3-phytol | C_38_H_68_O_2_ | 574.5563 | 278.2974 |
| 18:2-phytol | C_38_H_70_O2 | 576.5720 | 278.2974 |
| 18:1-phytol | C_38_H_72_O_2_ | 578.5876 | 278.2974 |
| 18:0-phytol | C_38_H_74_O_2_ | 580.6033 | 278.2974 |
| 20:0-phytol | C_40_H_78_O_2_ | 608.6346 | 278.2974 |

**Table S2.** Target list for Q-TOF MS/MS analysis of wax esters.

Wax esters form ammonium adducts [M + NH_4_]^+^ (top part) and produce protonated fatty acid ions [fatty acid + H]^+^ upon fragmentation (bottom part). Wax esters were quantified by product ion scanning of the [fatty acid + H]^+^ fragment. The mass of the respective alcohol moiety was calculated from the neutral loss between the parental ion and the product ion. This difference represents the dehydrated alcohol moiety [alcohol – H_2_O + NH_3_]. The collision energy was 15 V. I.S., internal standard.

| \| Parental Ions \|  \|  \| \| --- \| --- \| --- \| \| Wax ester  (main molecular species) \| Formula [M] \| [M + NH_4_]^+^  (*m/z*) \| \|  \| C_22_H_44_O_2_ \| 358.3680 \| \|  \| C_24_H_48_O_2_ \| 386.3993 \| \|  \| C_26_H_52_O_2_ \| 414.4306 \| \| 14:0-14:0ol \| C_28_H_56_O_2_ \| 442.4619 \| \|  \| C_30_H_60_O_2_ \| 470.4932 \| \| 16:0-16:0ol \| C_32_H_64_O_2_ \| 498.5245 \| \|  \| C_34_H_68_O_2_ \| 526.5558 \| \| 17:0-18:0ol (I.S.) \| C_35_H_70_O_2_ \| 540.5714 \| \| 18:0-18:0ol \| C_36_H_72_O_2_ \| 553.5871 \| \|  \| C_38_H_76_O_2_ \| 582.6184 \| \| 20:0-20:0ol \| C_40_H_80_O_2_ \| 609.6497 \| \|  \| C_42_H_84_O_2_ \| 638.6810 \| \|  \| C_44_H_88_O_2_ \| 666.7123 \| \|  \| C_46_H_92_O_2_ \| 694.7436 \| \|  \| C_48_H_96_O_2_ \| 722.7749 \| \| Product Ions \|  \|  \| \| Fatty Acid \| Formula [fatty acid] \| [fatty acid + H]^+^ (*m/z*) \| \| 12:0 \| C_12_H_24_O_2_ \| 217.2041 \| \| 14:0 \| C_14_H_28_O_2_ \| 245.2354 \| \| 16:0 \| C_16_H_32_O_2_ \| 273.2667 \| \| 17:0 (I.S.) \| C_17_H_34_O_2_ \| 271.2632 \| \| 18:0 \| C_18_H_36_O_2_ \| 301.2980 \| \| 20:0 \| C_20_H_40_O_2_ \| 313.3101 \| \| 22:0 \| C_22_H_44_O_2_ \| 341.3414 \| \| 24:0 \| C_24_H_48_O_2_ \| 369.3727 \| |  |
| --- | --- | --- | --- | --- | --- | --- | --- | --- | --- | --- | --- | --- | --- | --- | --- | --- | --- | --- | --- | --- | --- | --- | --- | --- | --- | --- | --- | --- | --- | --- | --- | --- | --- | --- | --- | --- | --- | --- | --- | --- | --- | --- | --- | --- | --- | --- | --- | --- | --- | --- | --- | --- | --- | --- | --- | --- | --- | --- | --- | --- | --- | --- | --- | --- | --- | --- | --- | --- | --- | --- | --- | --- | --- | --- | --- | --- | --- | --- | --- | --- | --- | --- |

**Table S3** Target list for the Q-TOF MS/MS analysis of hexadecanediol diesters.

Hexadecanediol diesters form ammonium adducts [M + NH_4_]^+^ in the positive mode (top). They were quantified by neutral loss scanning of the fatty acid [fatty acid – H_2_O + NH_3_] (bottom). The identity of the second fatty acid was obtained from the *m/z* of the remaining monoester peak. Note that unsaturated acyl groups were absent from the hexadecanediol diesters.

| Parental Ions |  |  |
| --- | --- | --- |
| Wax diester  (main molecular species) | Formula [M] | [M + NH_4_]^+^  (*m/z*) |
| di8:0 | C_32_H_62_O_4_ | 528.4992 |
| 8:0-10:0 | C_34_H_66_O_4_ | 556.5305 |
| di10:0 | C_36_H_70_O_4_ | 584.5618 |
| 10:0-12:0 | C_38_H_74_O_4_ | 612.5931 |
| di12:0, 10:0-14:0 | C_40_H_78_O_4_ | 640.6244 |
| 12:0-14:0 | C_42_H_82_O_4_ | 668.6557 |
|  | C_44_H_80_O_4_ | 690.6400 |
|  | C_44_H_82_O_4_ | 692.6557 |
|  | C_44_H_84_O_4_ | 694.6713 |
| di14:0, 12:0-16:0 | C_44_H_86_O_4_ | 696.6870 |
|  | C_46_H_84_O_4_ | 718.6713 |
|  | C_46_H_86_O_4_ | 720.6870 |
|  | C_46_H_88_O_4_ | 722.7026 |
| 14:0-16:0 | C_46_H_90_O_4_ | 724.7183 |
|  | C_48_H_88_O_4_ | 746.7026 |
|  | C_48_H_90_O_4_ | 748.7183 |
|  | C_48_H_92_O_4_ | 750.7339 |
| di16:0, 14:0-18:0 | C_48_H_94_O_4_ | 752.7496 |
| 16:0-18:0 | C_50_H_98_O_4_ | 780.7809 |
| Neutral Loss |  |  |
| Fatty Acid | Formula [fatty acid] | [fatty acid – H_2_O + NH_3_] |
| 8:0 | C_8_H_18_O_2_ | 143.1310 |
| 10:0 | C_10_H_20_O_2_ | 171.1623 |
| 12:0 | C_12_H_24_O_2_ | 199.1936 |
| 14:0 | C_14_H_28_O_2_ | 227.2249 |
| 16:3 | C_16_H_26_O_2_ | 249.2093 |
| 16:2 | C_16_H_28_O_2_ | 251.2249 |
| 16:1 | C_16_H_30_O_2_ | 253.2406 |
| 16:0 | C_16_H_32_O_2_ | 255.2562 |
| 18:3 | C_18_H_30_O_2_ | 277.2406 |
| 18:2 | C_18_H_32_O_2_ | 279.2562 |
| 18:1 | C_18_H_34_O_2_ | 281.2719 |
| 18:0 | C_18_H_36_O_2_ | 283.2875 |

**Table S4** ^1^H and ^13^C NMR chemical shift data for lipid X (diacyl-1,6-hexadecanediol)

^1^H and ^13^C NMR spectra were recorded at 700.43 MHz/176.12 MHz in CDCl_3_ at 300 K. ^13^C assignments are based on ^1^H,^13^C-HSQC, ^1^H,^13^C-HSQC-TOCSY and ^1^H,^13^C-HMBC. All chemical shifts are referenced to the residual solvent signal (δ_H_ = 7.26; δ_C_ = 77.16).

| ^1^H NMR data  Chemical shift / Coupling constant | |  | ^13^C NMR data  Chemical shift | |
| --- | --- | --- | --- | --- |
| Proton (signal form) | δ (ppm) / *J* (Hz) |  | Carbon | δ (ppm) |
| 1,6-hexadecanediol | |  |  |  |
| 1-H (t)^a^ | 4.04 / 6.7 |  | C-1 | 64.2 |
| 2-H (m)^a^ | 1.64-1.59 |  | C-2 | 28.6 |
| 3-H (m) | 1.36-1.29 |  | C-3 | 25.0 |
| 4-H (m) | 1.39-1.32 |  | C-4 | 25.8 |
| 5-H (m) | 1.55-1.50 |  | C-5 | 34.1 |
| 6-H (m) | 4.89-4.84 |  | C-6 | 73.9 |
| 7-H (m) | 1.53-1.47 |  | C-7 | 34.0 |
| 8-H (m) | 1.31-1.24 |  | C-8 | 25.4 |
| 9-H (m) | 1.28-1.24 |  | C-9 | 29.6 |
| Ester-bound fatty acids | |  |  |  |
|  |  |  | CO | 173.9^O-1^, 173.6^O-6^ |
| α-H @ O-1 (t) | 2.28 / 7.5 |  | C-α^O-1^ | 34.5 |
| α-H @ O-6 (t) | 2.27 / 7.4 |  | C-α^O-6^ | 34.7 |
| β-H (m) | 1.65-1.59 |  | C-β | 25.1 |
| γ-H (m) | 1.33-1.29 |  | C-γ | 29.3 |
| Further aliphatic signals | |  |  |  |
| CH_2_-groups | 1.31-1.24^b^ |  | CH_2_-groups | 29.9 ^…^ 29.3^b^ |
| CH_3_-CH_2_-CH_2_ (m) | 1.28-1.23 |  | CH_3_-CH_2_-CH_2_ | 31.9 |
| CH_3_-CH_2_ (m) | 1.33-1.26 |  | CH_3_-CH_2_ | 22.6 |
| CH_3_-CH_2_ (t) | 0.88 / 7.1 |  | CH_3_-CH_2_ | 14.2 |
| ^a^t, triplet; m, multiplet. | | | | |
| ^b^ no specified assignment possible due to too many overlapping signals. | | | | |
